# Supplementary material for: Ribosomal DNA promoter recognition is determined in vivo by cooperation between UBTF1 and SL1 and is compromised in the UBTF-E210K neuroregression syndrome
Source: PLoS Genet. 2022 Feb 9;18(2):e1009644. doi: 10.1371/journal.pgen.1009644 (PMC8863233; doi:10.1371/journal.pgen.1009644)
Supplement: S1 Text — (DOCX) [file pgen.1009644.s001.docx]

**S1 Text. The Taf1B gene is essential for mouse development beyond early blastula.**

Mouse lines carrying a targeted “Knockout First” insertion in the gene for TAF1B (Taf68), were established and these crossed to remove the *ß-Gal* and *Neo* cassette insertion, generating lines carrying lox sites flanking exons 4 and 5 of *Taf1b* (S1A and S1B Fig). Subsequent recombination of these lox sites inactivated the *Taf1b* gene, (S1C Fig), see Supplementary Materials and Methods for more detail. Mice heterozygous for the *taf1b^∆^* allele were found to be both viable and fertile and the null-allele was propagated at near Mendelian frequency (S1 Table). However, no *taf1b^∆/∆^* homozygous offspring (pups) were identified and genotyping of embryos detected no *taf1b^∆/∆^* homozygotes at stages 6.5 and later. In contrast, four *taf1b^∆/∆^* embryos were detected at 3.5 dpc, though only one of these displayed a recognizable blastula morphology (S1D and S1E Fig). It was concluded that *taf1b* was essential for mouse development beyond blastula but that maternal Taf1B mRNA or protein, or simply ribosome availability may have been sufficient to support development beyond the morula stage. This is fully consistent with the previous data for inactivation of the TBP gene *tbp*/*gtf2d* [1] and suggests the interesting possibility that the effects of TBP-loss on early development could in large part be due to inactivation of RPI transcription. In support of this possibility, the SL1 complex is known to be generally less abundant than the RPII/PolII TFIID complex [2] and so could be limiting for embryo growth. Further, inactivation of the genes for the RPI factors UBTF (*Ubtf)* and RRN3/TIF1A (*Rrn3*) arrest mouse development during early cleavage stages [3, 4]. A similar argument could be made for inactivation of RPIII/PolIII transcription since loss of the Brf1 subunit of the TFIIIB complex also causes developmental arrest during early cleavage stages [5]. We conclude that the maternal protein translation machinery is limiting in the cleavage embryo and must be replenished by zygotic expression to allow further development.

**References**

1. Martianov I, Viville S, Davidson I. RNA polymerase II transcription in murine cells lacking the TATA binding protein. Science. 2002;298(5595):1036-9.

2. Comai L, Tanese N, Tjian R. The TATA-binding protein and associated factors are integral components of the RNA polymerase I transcription factor, SL1. Cell. 1992;68:965-76.

3. Hamdane N, Stefanovsky VY, Tremblay MG, Nemeth A, Paquet E, Lessard F, et al. Conditional inactivation of Upstream Binding Factor reveals its epigenetic functions and the existence of a somatic nucleolar precursor body. PLoS Genetics. 2014;10(8):e1004505.

4. Herdman C, Mars JC, Stefanovsky VY, Tremblay MG, Sabourin-Felix M, Lindsay H, et al. A Unique Enhancer Boundary Complex on the Mouse Ribosomal RNA Genes persists after loss of Rrn3 or UBF and the Inactivation of RNA Polymerase I Transcription. PLoS Genetics. 2017;13(7):e1006899.

5. Liko D, Mitchell L, Campbell KJ, Ridgway RA, Jones C, Dudek K, et al. Brf1 loss and not overexpression disrupts tissues homeostasis in the intestine, liver and pancreas. Cell Death Differ. 2019;26(12):2535-50.
