# Supplementary material for: Ribosomal DNA promoter recognition is determined in vivo by cooperation between UBTF1 and SL1 and is compromised in the UBTF-E210K neuroregression syndrome
Source: PLoS Genet. 2022 Feb 9;18(2):e1009644. doi: 10.1371/journal.pgen.1009644 (PMC8863233; doi:10.1371/journal.pgen.1009644)
Supplement: S1 Table — (DOCX) [file pgen.1009644.s002.docx]

**S1 Table**

| \| Age (dpc) \| Total No. \| *Taf1b^wt/wt^* \| *Taf1b^∆/wt^* \| *Taf1b^∆/∆^* \| \| --- \| --- \| --- \| --- \| --- \| \| 3.5 \| 20 \| 3 (15%) \| 13 (65%) \| 4 (20%) \| \| 6.5 \| 23 \| 12 (52%) \| 11 (48%) \| 0 (0%) \| \| 7.5 \| 15 \| 6 (40%) \| 9 (60%) \| 0 (0%) \| \| 8.5 \| 35 \| 8 (23%) \| 27 (77%) \| 0 (0%) \| \| 9.5 \| 16 \| 4 (25%) \| 12 (75%) \| 0 (0%) \| \| Pups \| 112 \| 35 (31%) \| 77 (69%) \| 0 (0%) \| |
| --- | --- | --- | --- | --- | --- | --- | --- | --- | --- | --- | --- | --- | --- | --- | --- | --- | --- | --- | --- | --- | --- | --- | --- | --- | --- | --- | --- | --- | --- | --- | --- | --- | --- | --- | --- |
