# Supplementary figures and images for: Ribosomal DNA promoter recognition is determined in vivo by cooperation between UBTF1 and SL1 and is compromised in the UBTF-E210K neuroregression syndrome
Source: PLoS Genet. 2022 Feb 9;18(2):e1009644. doi: 10.1371/journal.pgen.1009644 (PMC8863233; doi:10.1371/journal.pgen.1009644)

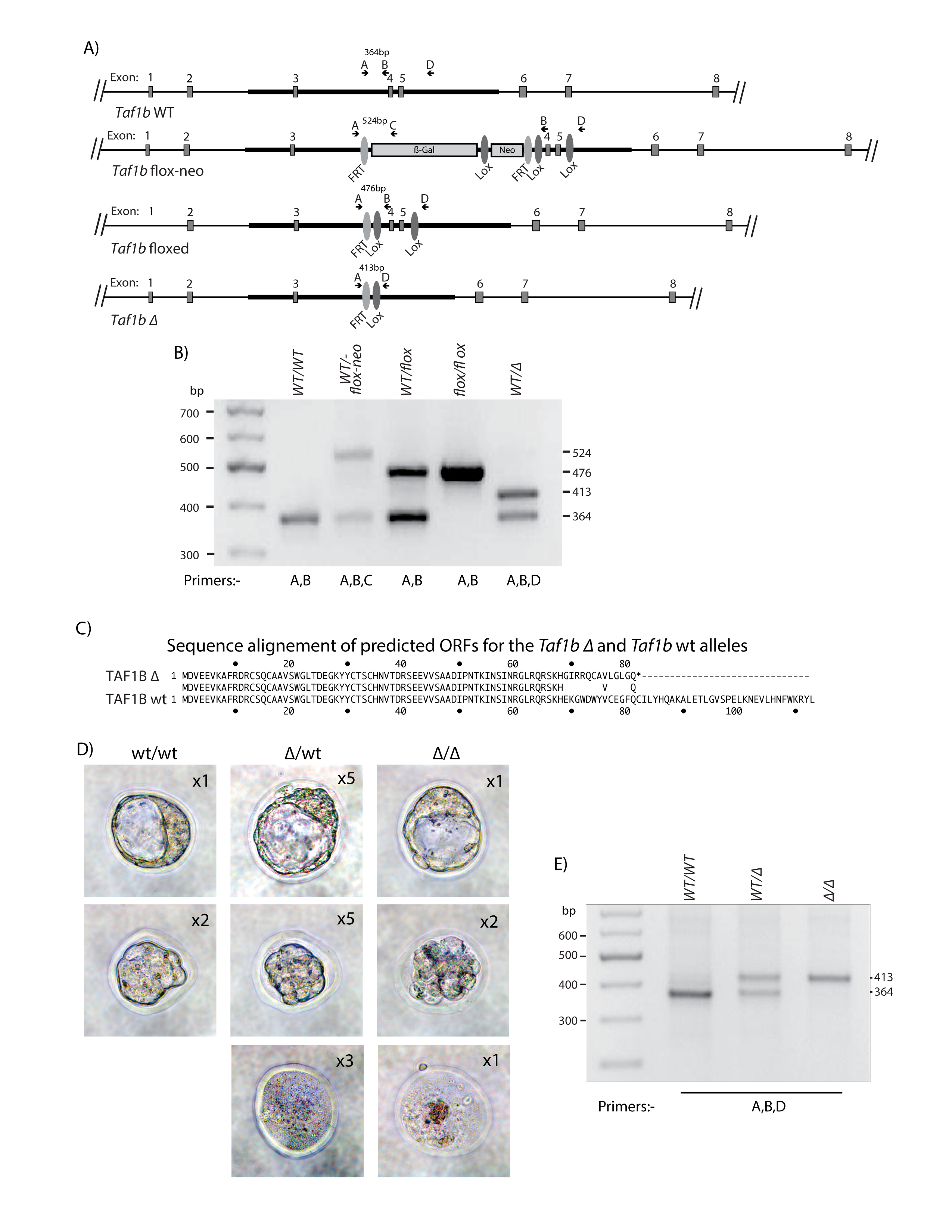

Supplement: S1 Fig — A) Organisation of the first 8 exons of the mouse Taf1b gene (Taf1bwt), and the “flox-neo” insertion, “floxed” and alleles indicating the position of inserted FRT and Lox sites and the inactivated (Taf1bΔ) allele after Lox site recombination to delete exons 4 and 5. The positions of genotyping primers A to D are also indicated. B) Examples of mouse PCR genotyping. C) alignment of the N-terminal sequence of wild type TAF1B with the predicted residual TAF1B peptide encoded by the Taf1bΔ allele. D) Typical images of mouse embryos at 3.5 dpc derived from Taf1bwt/Δ mouse crosses. The corresponding genotypes and numbers of embryos in each class are indicated, see also S1 Table. E) Embryo phenotyping using primers A, B and D shown in panel A. (TIF) [file pgen.1009644.s003.tif]

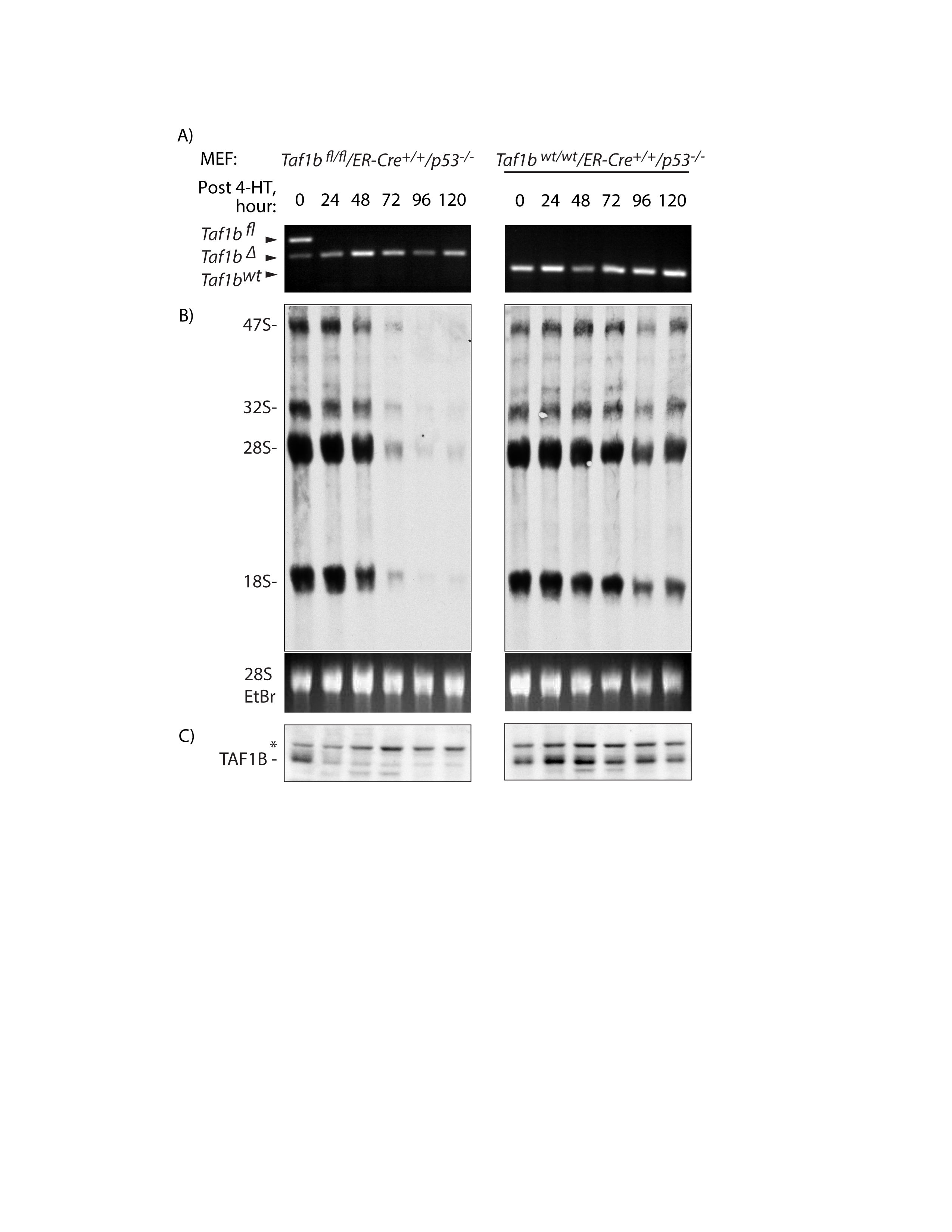

Supplement: S2 Fig — Conditional Taf1bfl/fl/p53-/-/ERcre+/+ and control Taf1bwt/wt/p53-/-/ERcre+/+ MEFs were treated with 50 nM 4-hydroxy-tamoxifen (4-HT) for 4h (4-HT pulse) before removing 4-HT by a change of the culture medium. 47S pre-rRNA synthesis was then determined by [3H]-uridine RNA metabolic labelling. The panels show the parallel time course analyses post 4-HT treatment of; A) Taf1b inactivation, B) [3H]-rRNA labelling versus steady state 28S rRNA, and C) TAF1B protein depletion. The 47S pre-rRNA and rRNA processing products are indicated in B. The “*” in C indicates a non-specific antibody interaction that serves as a loading control. (TIF) [file pgen.1009644.s004.tif]

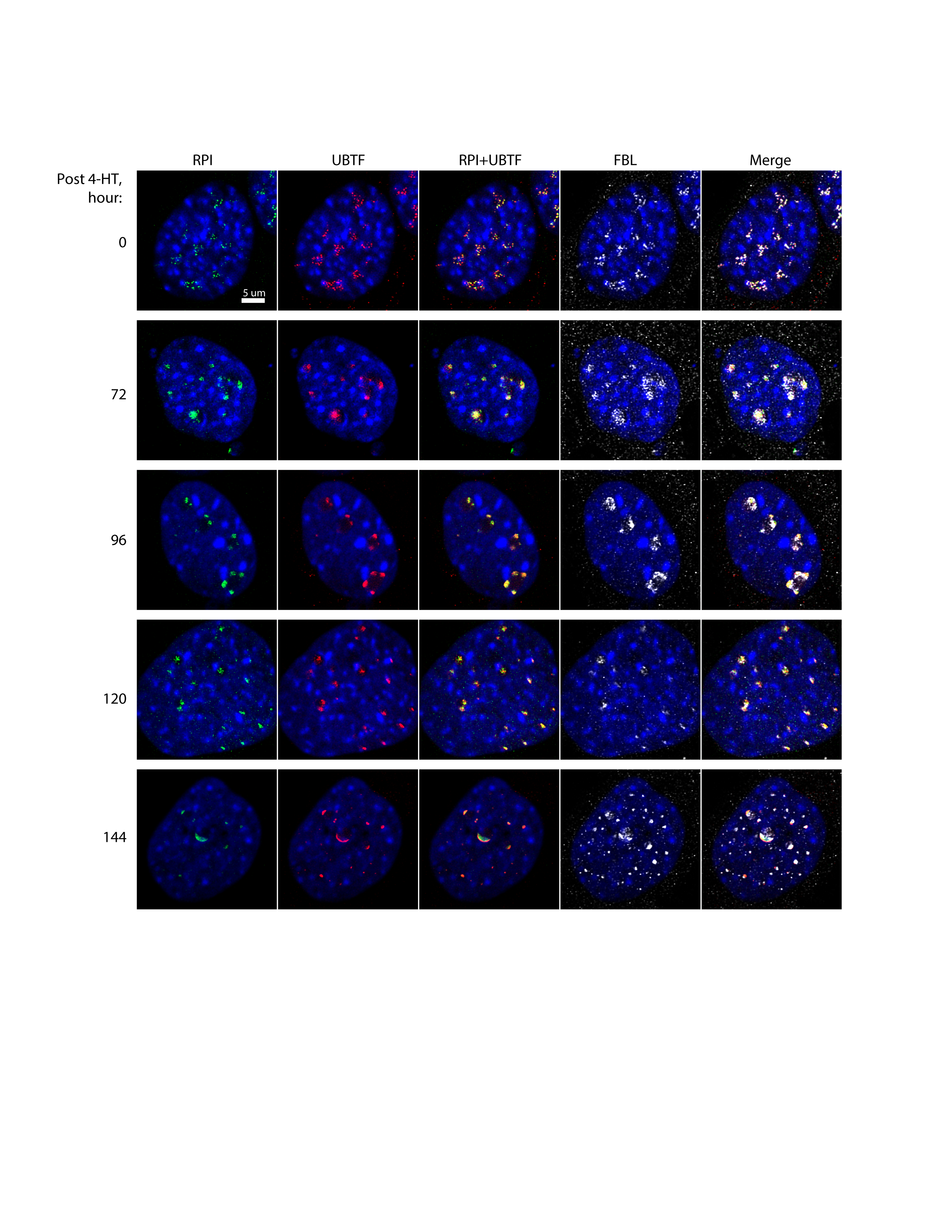

Supplement: S3 Fig — Single confocal image planes are shown as for each factor and as merged overlays each with DAPI staining of DNA. (TIF) [file pgen.1009644.s005.tif]

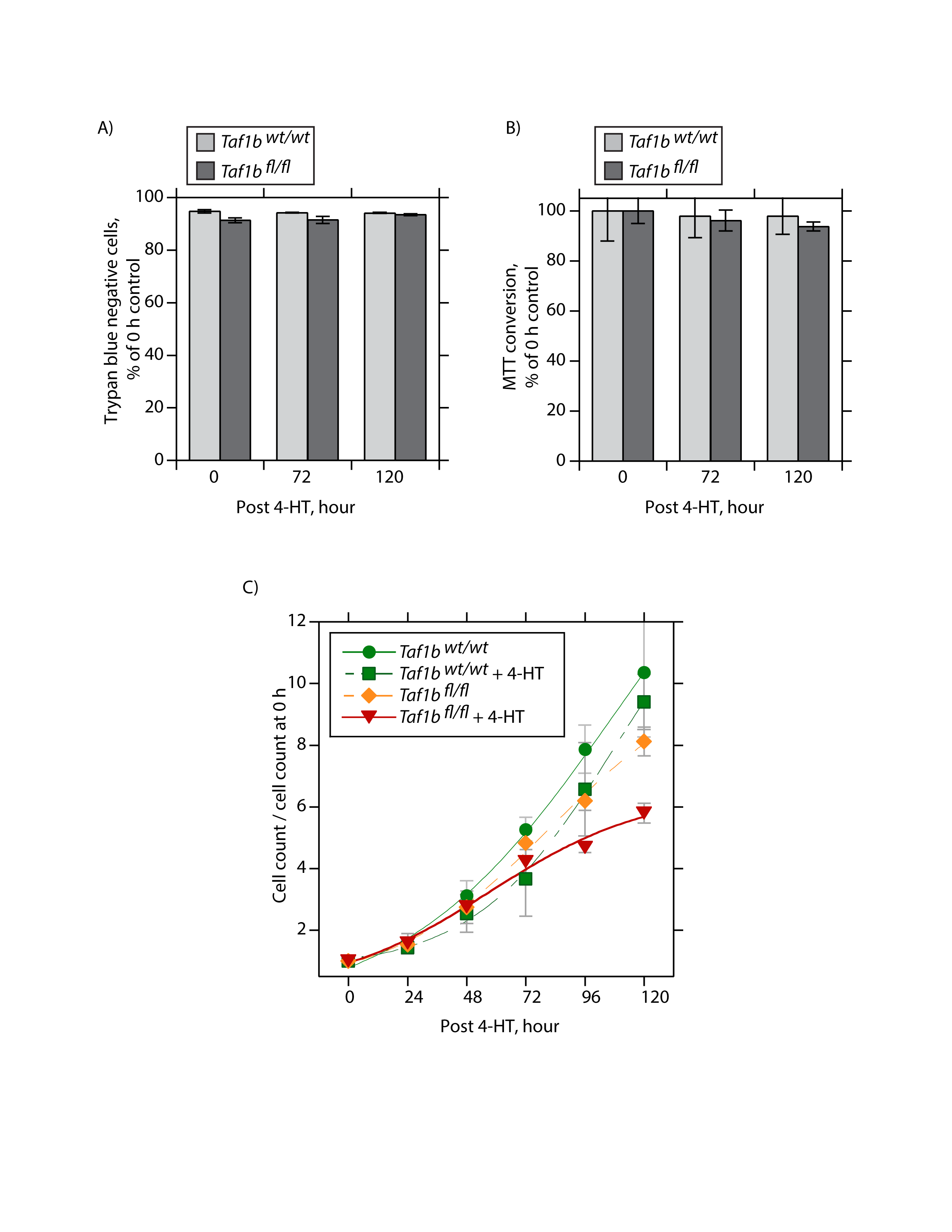

Supplement: S4 Fig — A) Trypan blue exclusion and B) MTT conversion assays for cell viability, and C) cell proliferation at different time points post 4-HT or mock treatment were determined in quadruplicate for Taf1bfl/fl/p53-/-/ERcre+/+ and Taf1bwt/wt/p53-/-/ERcre+/+MEFs. (TIF) [file pgen.1009644.s006.tif]

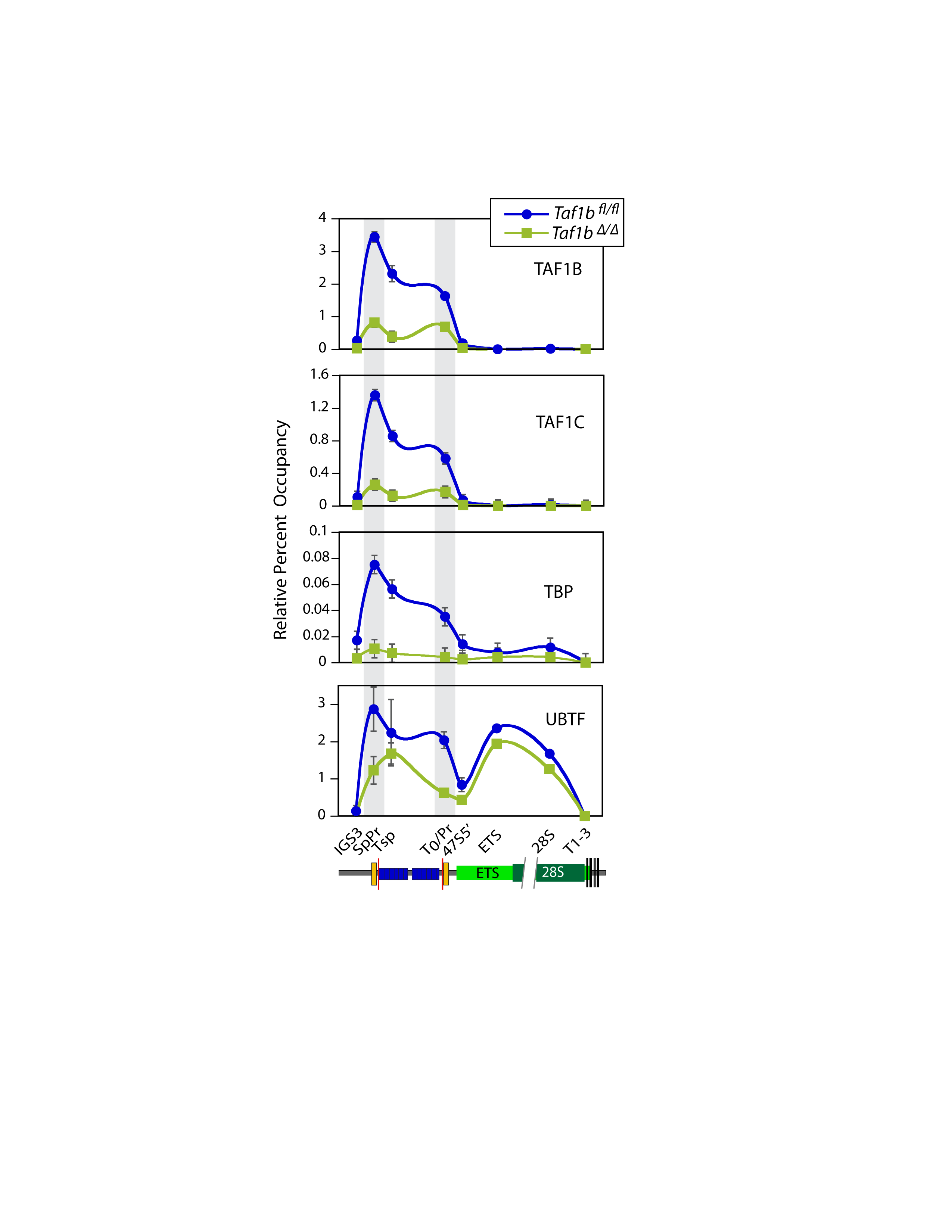

Supplement: S5 Fig — The data for TAF1B and UBTF derive from 3 ChIP biological replicas and for TAF1C and TBP a single ChIP each analyzed by qPCR in triplicate. (TIF) [file pgen.1009644.s007.tif]

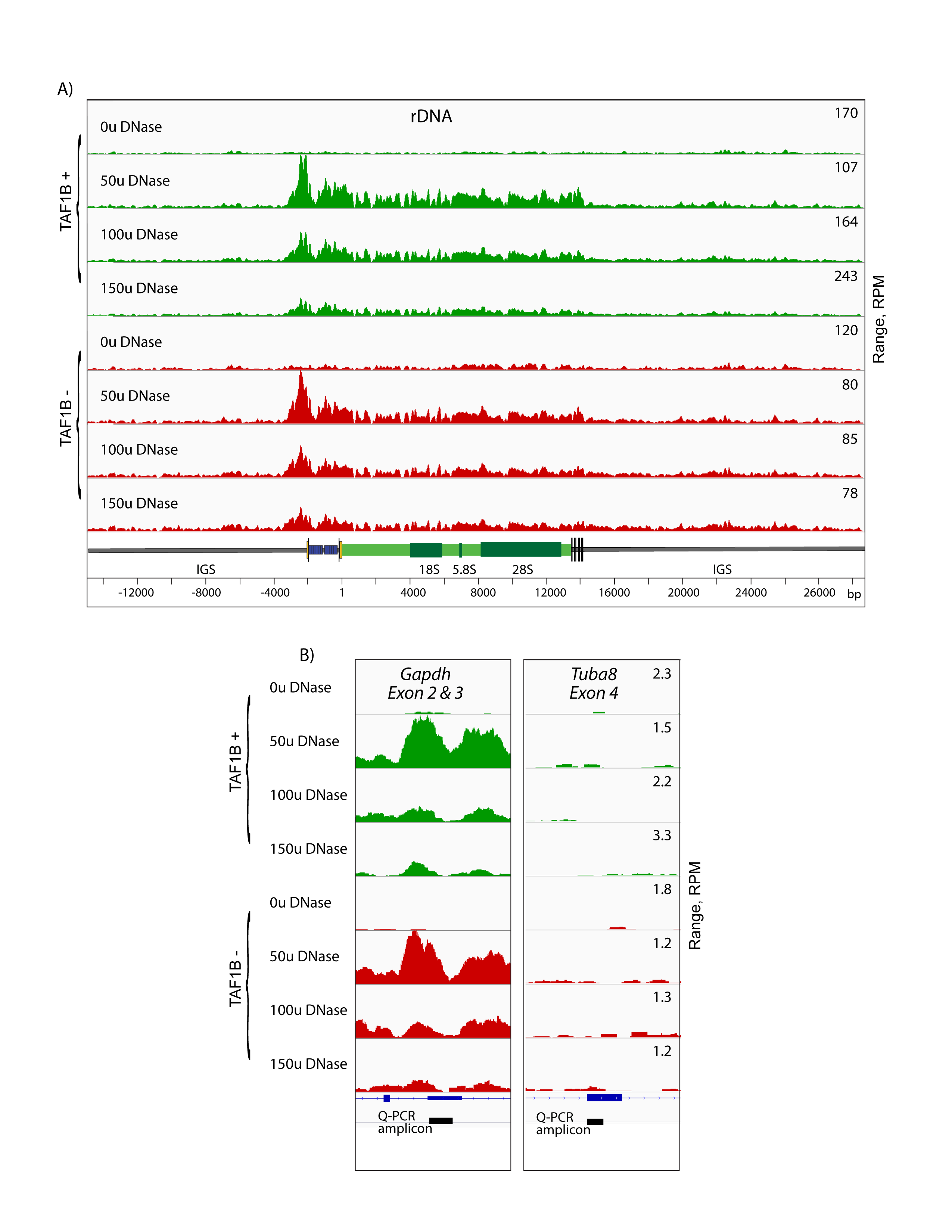

Supplement: S6 Fig — A) Mapping of raw sequencing data obtained from nuclei treated with increasing concentrations of DNase I (0 to 150 units) across the rDNA, see Materials and Methods. B) Same data sets were also mapped across the active euchromatic GAPDH and inactive heterochromatic Tub8a genes. “Q-PCR amplicons” indicate the Gapdh and Tub8a gene regions used to select appropriate DNaseI digestion to discriminate hyper-accessible gene regions. (TIF) [file pgen.1009644.s008.tif]

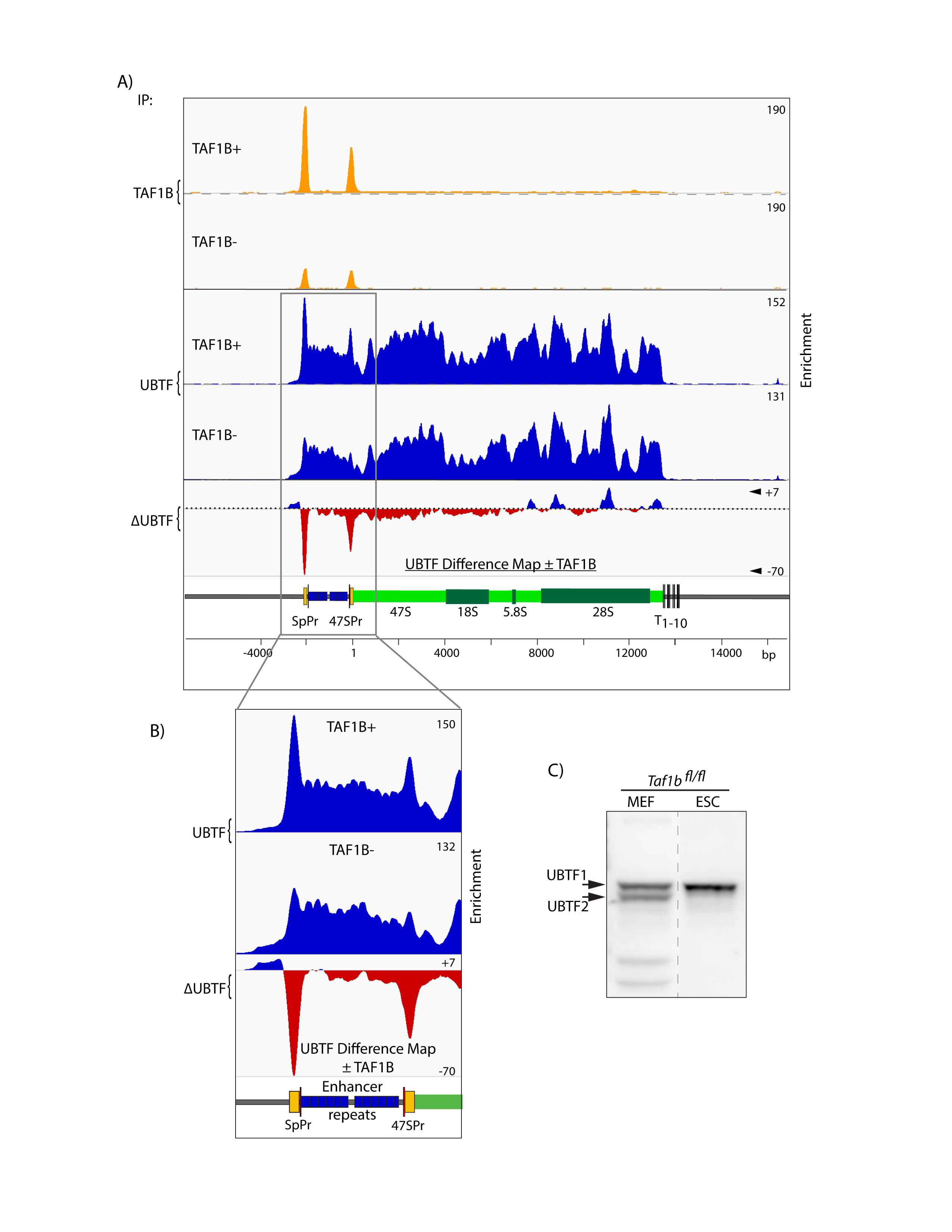

Supplement: S7 Fig — A) DChIP-Seq analysis of TAF1B and UBTF occupancy across the rDNA repeat before (TAF1B+) and 3 days after Taf1b inactivation (TAF1B-). ΔUBTF indicates the difference map of UBTF occupancy after TAF1B depletion minus the occupancy before TAF1B depletion. B) Magnified view of the DChIP mapping in A showing detail over the promoter and enhancer regions. C) Comparative Western blots of UBTF from TAF1B conditional MEFs (Taf1bfl/fl/p53-/-/ERcre+/+) and mESCs (Taf1bfl/fl/ERcre+/+), showing the presence of both UBTF1 and UBTF2 variants are expressed in the MEFs but only the UBTF1 variant is expressed in the mESCs. (TIF) [file pgen.1009644.s009.tif]

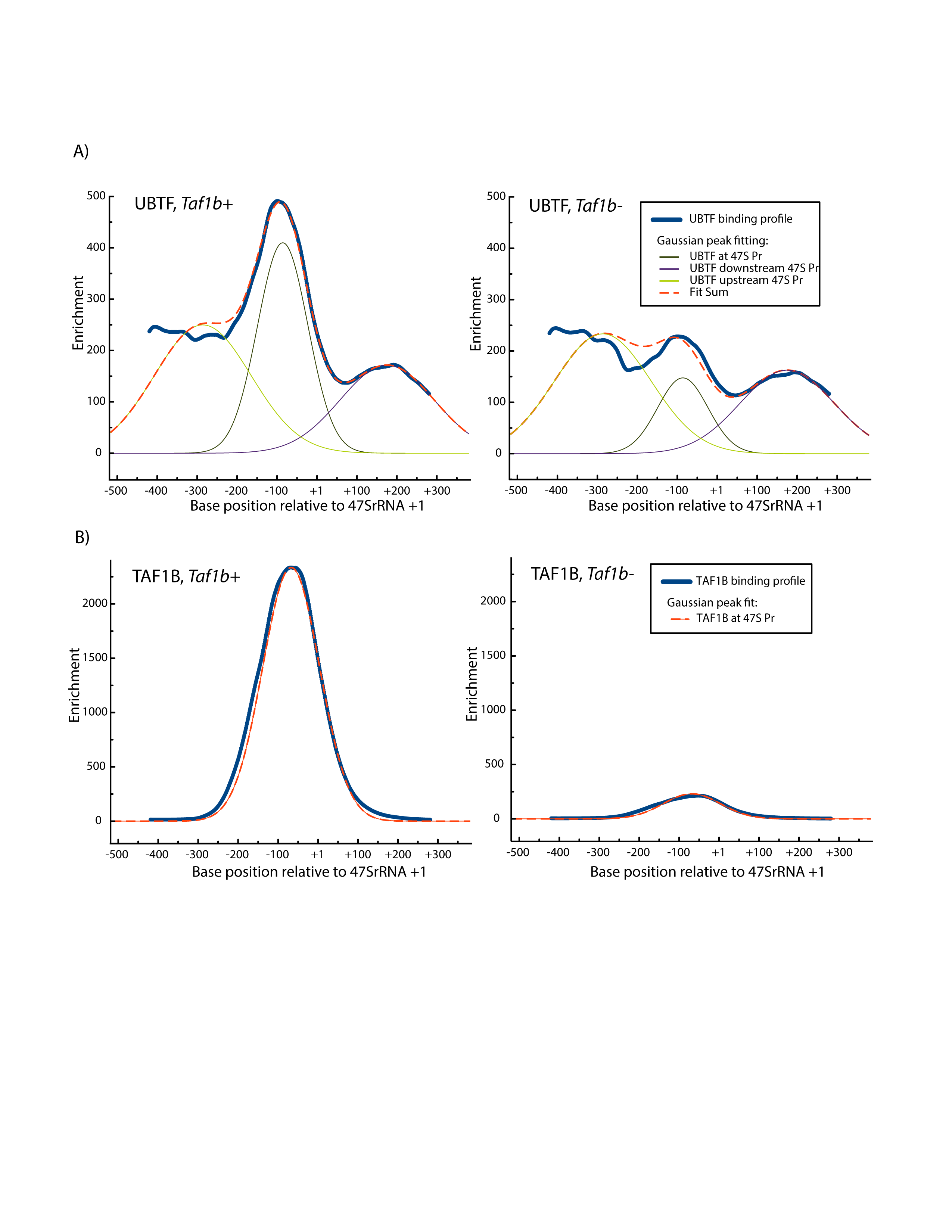

Supplement: S8 Fig — A) and B) respectively show examples of UBTF and TAF1B enrichment profiles over the 47S promoter region before and after Taf1b inactivation are shown, (dark blue line), and the best Gaussian peak fits to these profiles (dashed red line). In the case of TAF1B the profile closely followed a single Gaussian peak from which both the position and relative occupancy were determined. Since UBTF was present not only at the promoter but also over the adjacent regions, curve fits were made using three Gaussians peaks, and the central one used to estimate relative occupancy. (TIF) [file pgen.1009644.s010.tif]

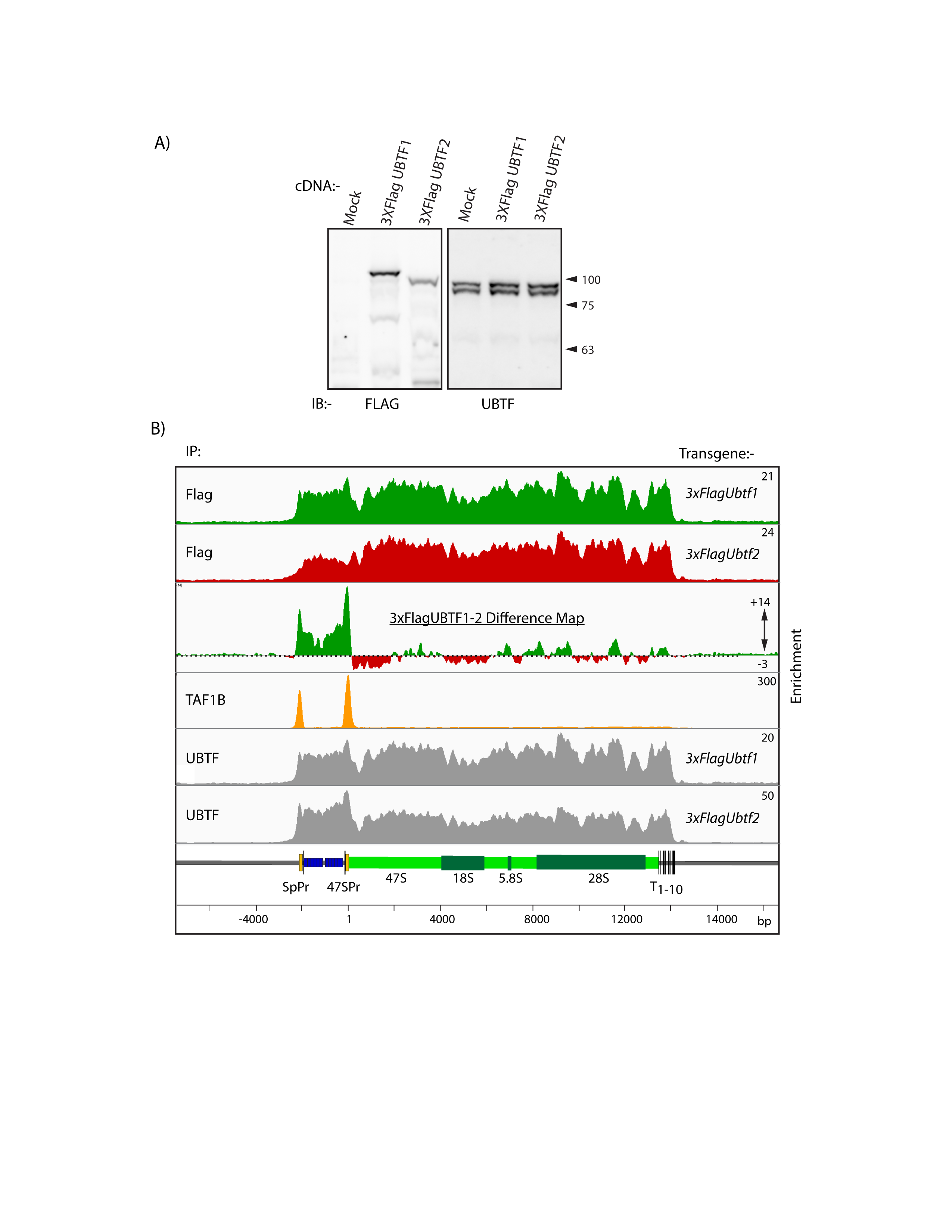

Supplement: S9 Fig — A) Expression of exogenous 3xFlag-UBTF1 and UBTF2 in NIH3T3 MEFs. Total cell protein extracts from Mock, 3xFlag-UBTF1 or 3xFlag-UBTF2 transfected cells were analyzed by Western blot using either anti-Flag (αFlag), left hand panel) or anti-UBTF (UBTF) antibodies to detect total UBTF (right hand panel). B) DChIP-Seq mapping profiles of the exogenously 3xFlag-UBTF1 or 3xFlag-UBTF2 (Flag) expressed in NIH3T3 MEFs (as in Fig 4) and the total endogenous UBTF profiles ChIPped from the same chromatin preparations. (TIF) [file pgen.1009644.s011.tif]

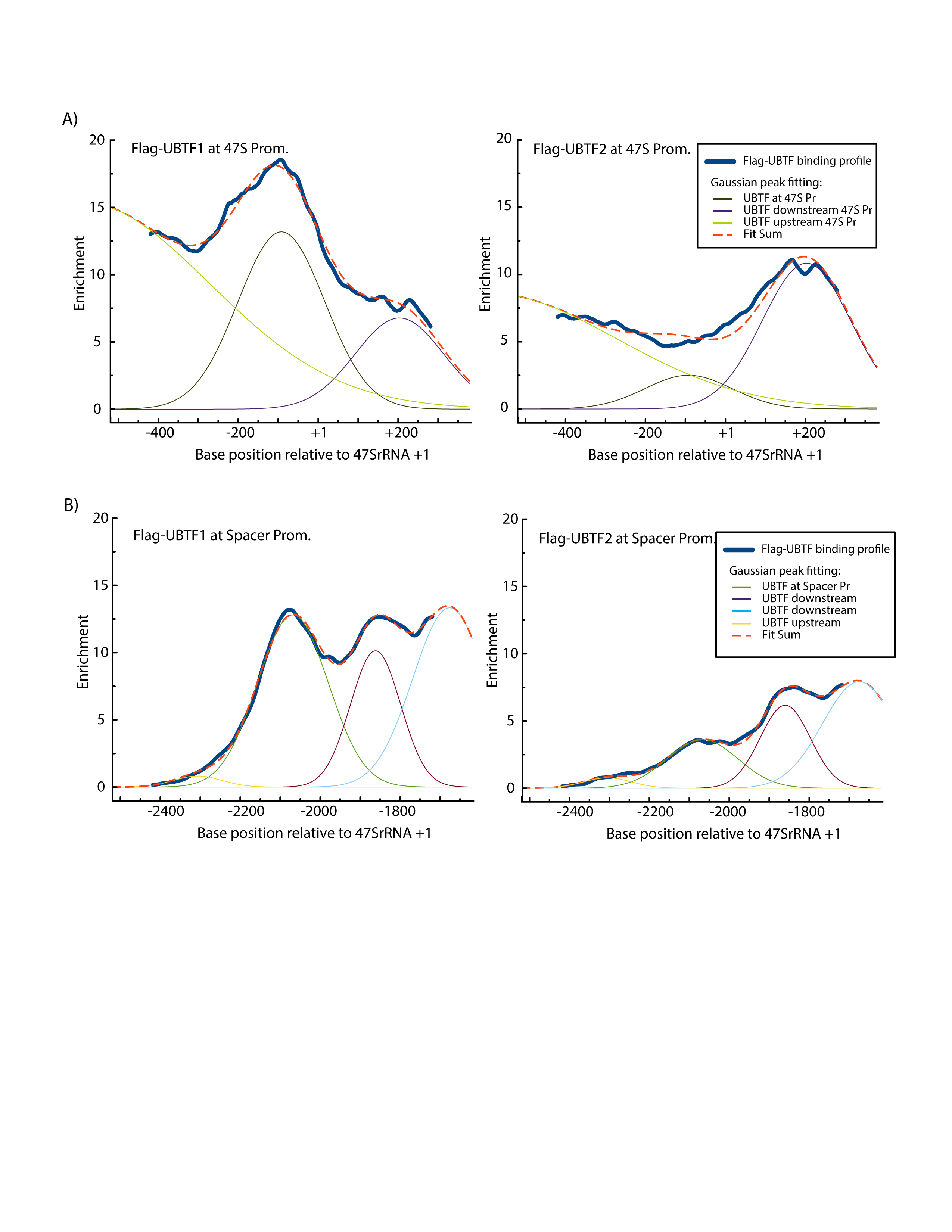

Supplement: S10 Fig — A) and B) Show examples of Flag-UBTF1 and Flag-UBTF2 enrichment profiles respectively over the 47S and Spacer promoter regions, (dark blue line). The best Gaussian peak fits to these profiles are shown (dashed red line), as are the individual Gaussian peaks used to estimate relative promoter occupancy. Since UBTF was present not only at each promoter but also over adjacent regions, curve fits were made using three, or in the case of the Spacer promoter four, Gaussians peaks. (TIF) [file pgen.1009644.s012.tif]

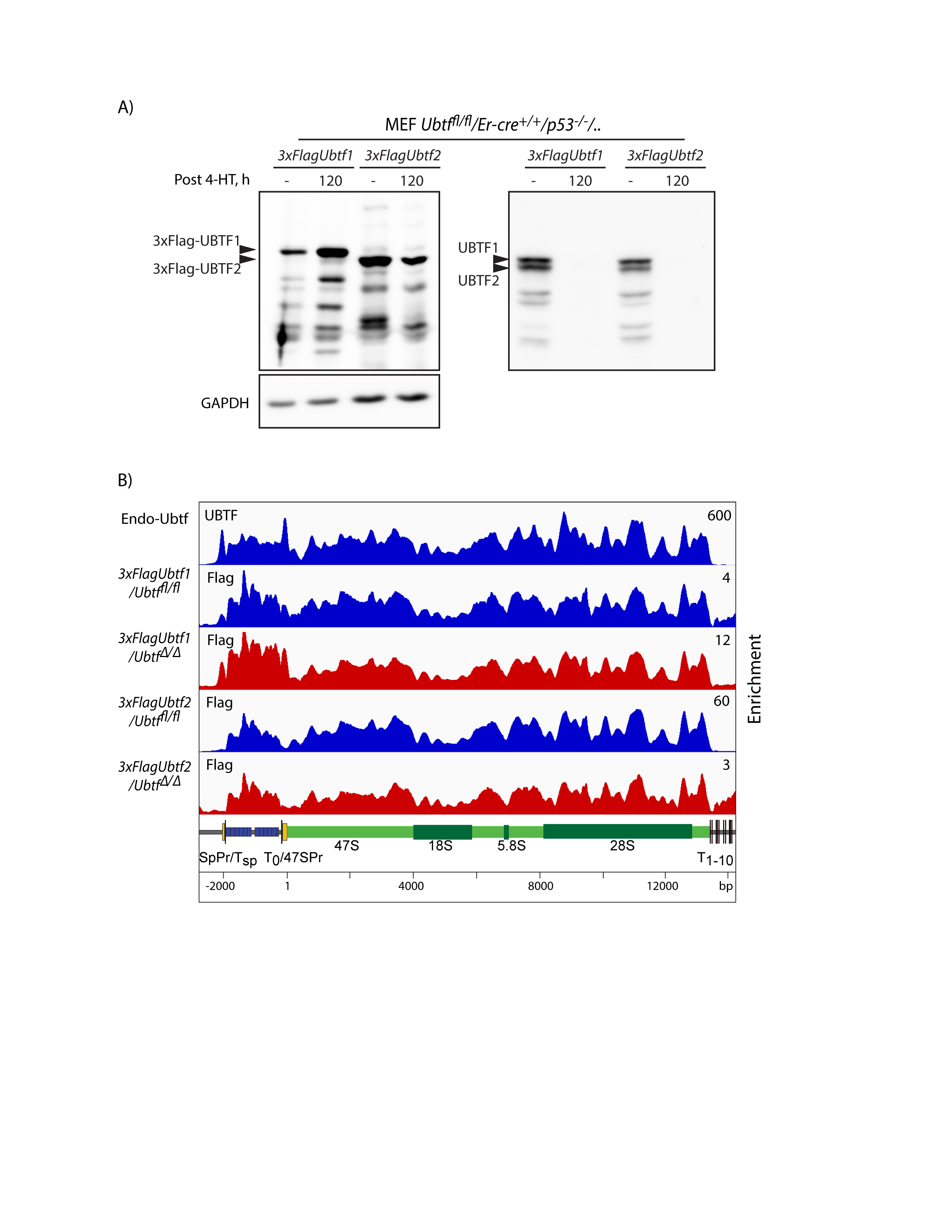

Supplement: S11 Fig — A) expression of 3Flag-UBTF1 and 2 and endogenous UBTF in conditional MEFs before and after UBTF deletion as revealed by Western blot using anti-Flag and anti-UBTF antibodies. B) D-ChIP mapping of UBTF forms across the full rDNA transcription unit as in Fig 5. As reference, the upper track shows mapping of the endogenous UBTF shown in Fig 3. (TIF) [file pgen.1009644.s013.tif]

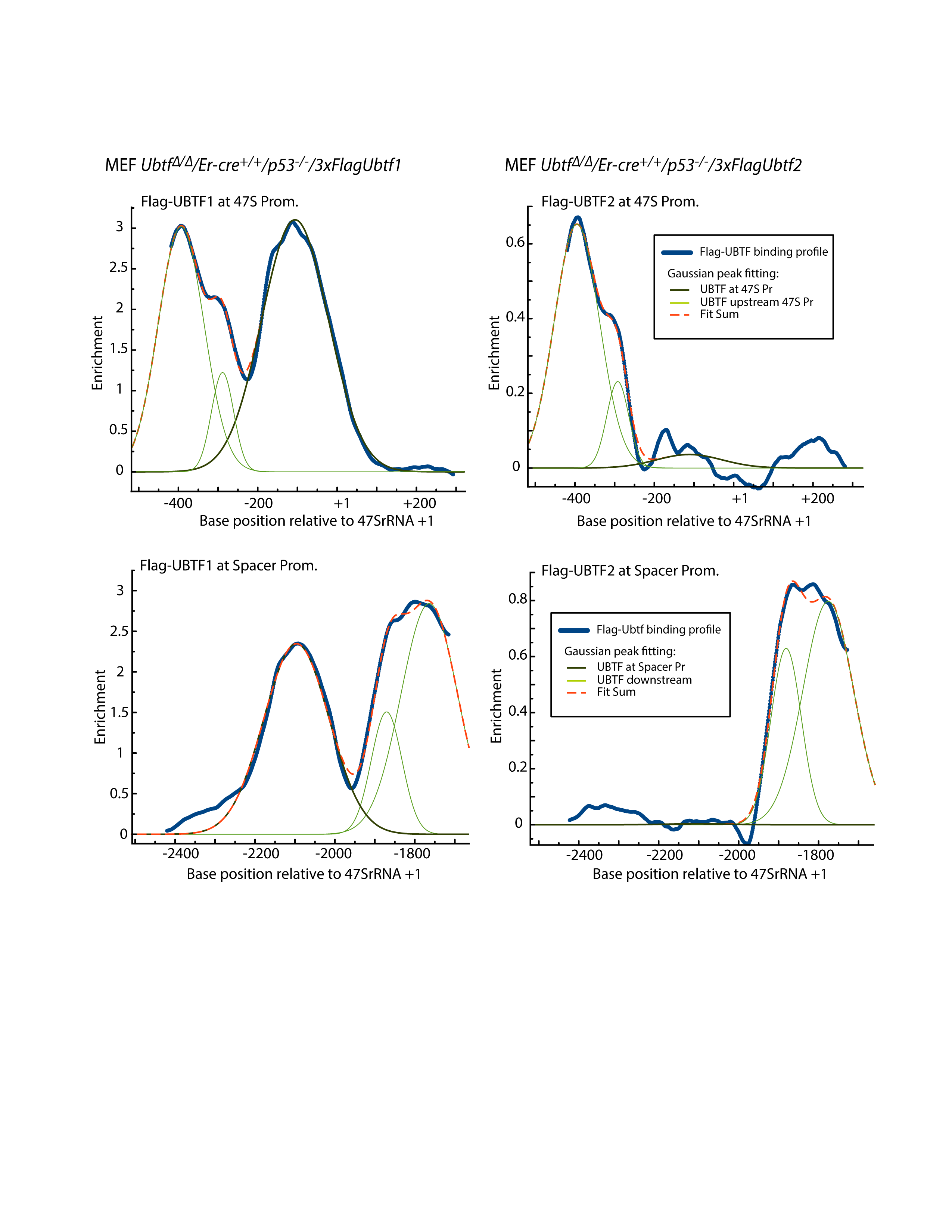

Supplement: S12 Fig — The area of Flag-UBTF1 or 2 peaks at each promoter was normalized to the summed area adjacent Flag-UBTF peaks to allow comparisons between profiles. Gaussian peak positions were maintained constant between analyses. (TIF) [file pgen.1009644.s014.tif]

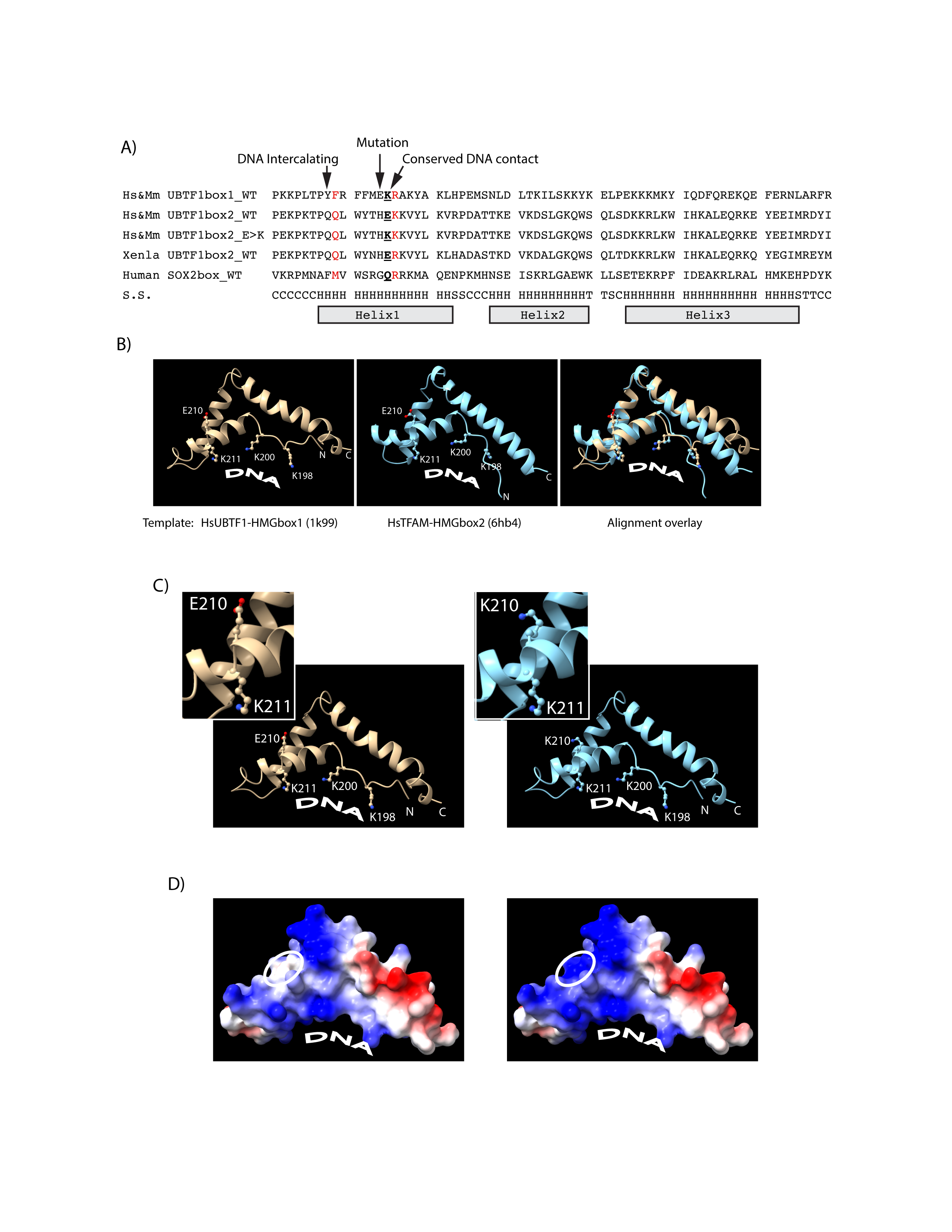

Supplement: S13 Fig — A) Sequence alignment of the HMGboxes 1 and 2 of human and mouse UBTF1 (NM_014233–2, NP_035681) with HMGbox2 of Xenopus laevis UBTF1a (CAA42523.1) and the HMGbox of SOX2 (P48431). The positions of the predicted DNA intercalating residue, the E210K mutation and the adjacent conserved basic DNA contacting residue are indicated as are the positions of the α-helical segments. B) Comparative molecular modelling of UBTF HMGbox2 using as templates the structures 1k99 (human UBTF HMGbox1) and 6hb4 (human mitochondrial transcription factor A, TFAM). The two predicated structures were generated by SWISS-MODEL [51] and are shown individually and as an aligned overlay generated in ChimeraX-1.1.1 [52]. Comparison of these structures using the Matchmaker routine in ChimeraX-1.1.1 revealed an RMSD of 1.215 Å over 41 of 72 alpha-carbons, including those of helix 1 affected by the E210K mutation. C) The predicted positions and orientations of the E210 and K210 residues within the HMGbox2 of UBTF1 are shown relative to the adjacent conserved basic residue at position 211, which is a lysine in UBTF/UBTF. The likely other DNA minor groove contacting residues K198 and K200 are also shown. D) The predicted surface electrostatic potential of the wild type, left, and the mutant, right, HMGbox2. Blue indicates a positive and red a negative potential. Position of changes in surface potential due to the E210K mutation are enclosed by an ellipse. (TIF) [file pgen.1009644.s015.tif]

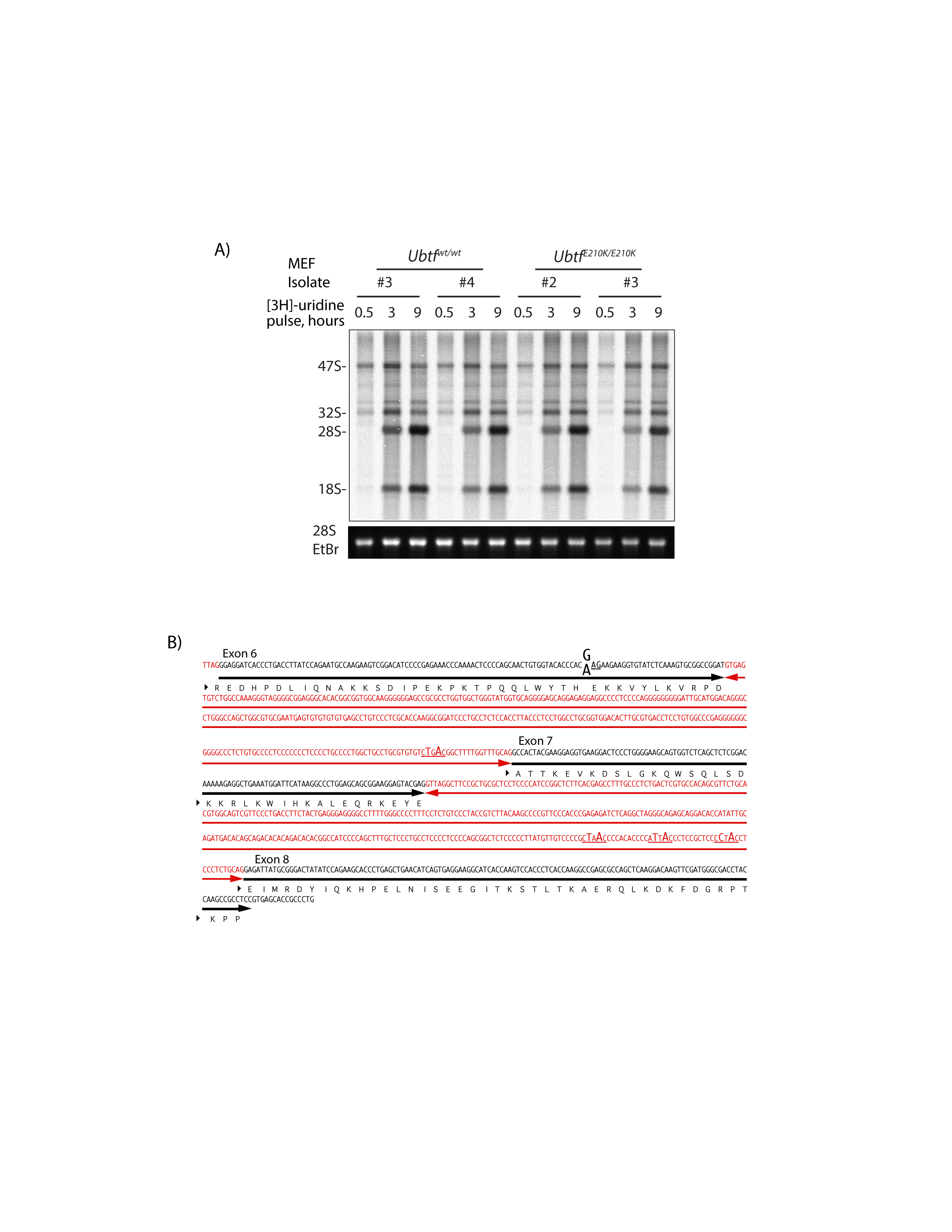

Supplement: S14 Fig — A) RNA metabolic pulse labelling to reveal 47S pre-rRNA synthesis and processing products in UbtfE210k/E210K knock-in and wild type Ubtfwt/wt MEFs. Gel fractionation of RNA after increasing labelling times is shown for two individual (numbered) MEF isolates. B) DNA base sequence of the differentially spliced region of mouse Ubtf gene showing coding exon 6, the differentially spliced coding exon 7 and coding exon 8 in black and the intervening introns in red (taken from GRCm38:11:102303960:102320342). The position of the G>A gene mutation, the cause of the E210K change in the UBTF protein, is indicated as are the potential splice branch sites in the intervening introns that most closely fit the yTnAy consensus [53]. (TIF) [file pgen.1009644.s016.tif]

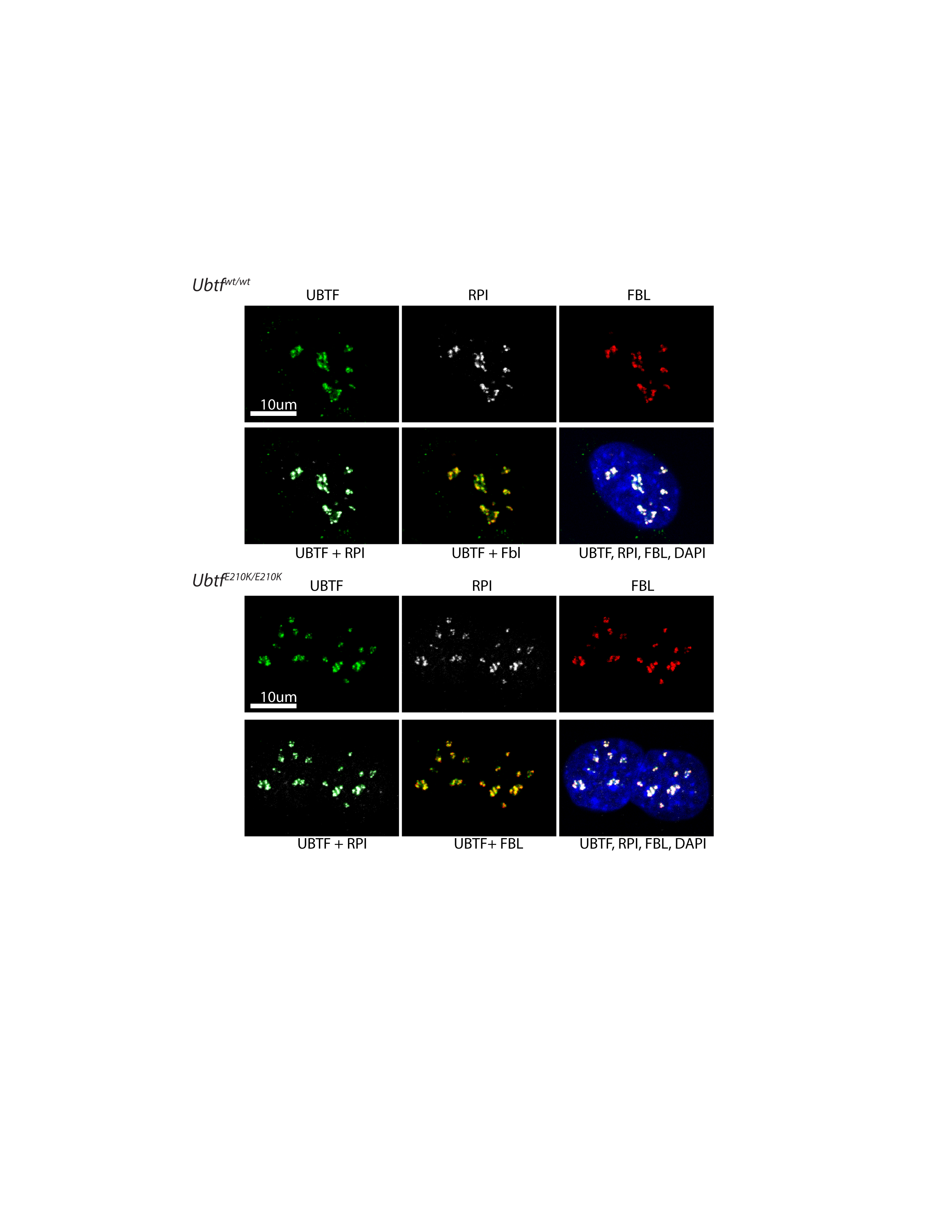

Supplement: S15 Fig — (TIF) [file pgen.1009644.s017.tif]

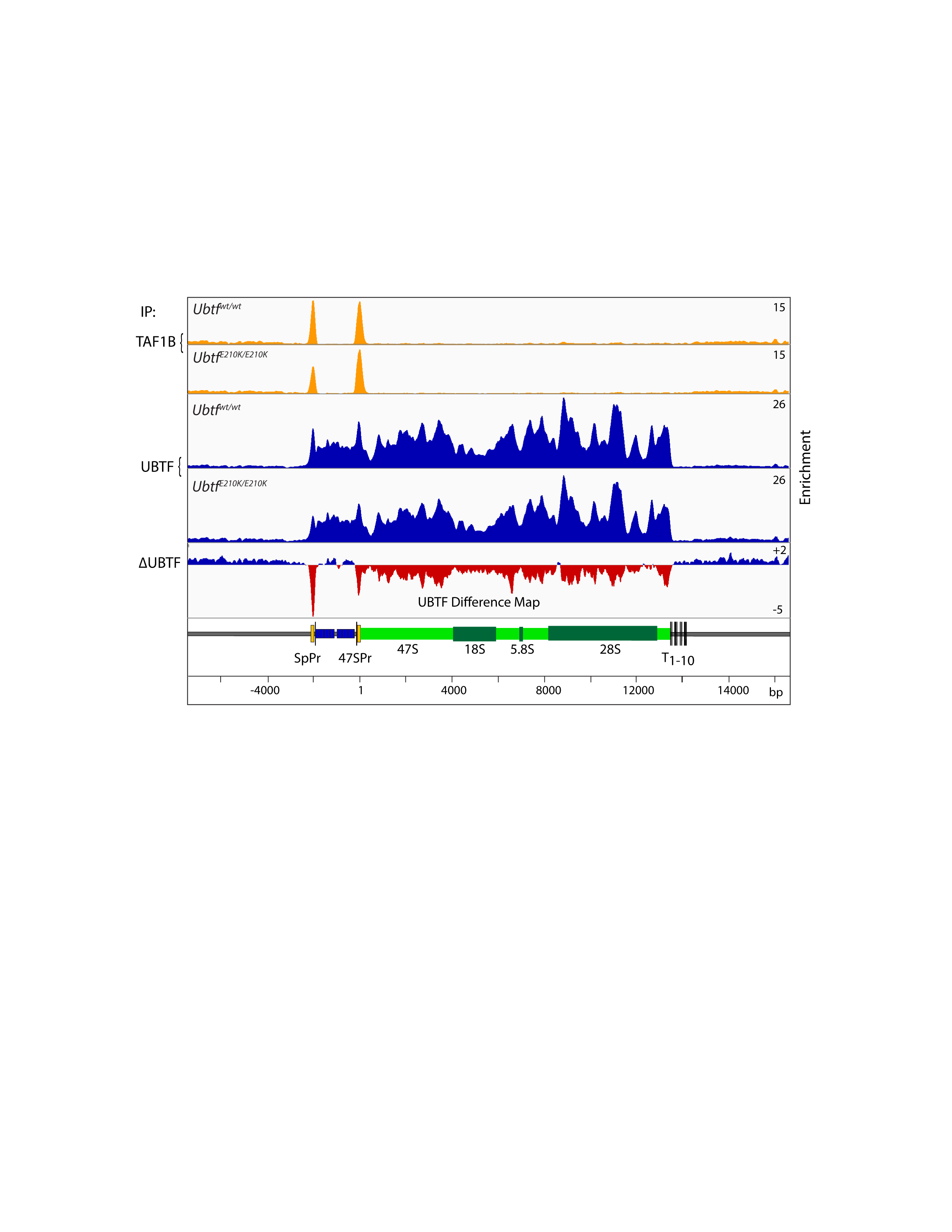

Supplement: S16 Fig — TAF1B and UBTF mapping are also shown for reference. (TIF) [file pgen.1009644.s018.tif]
